# Evaluation of Major Physical and Mechanical Properties of Trembling Aspen Lumber

**DOI:** 10.3390/ma17122952

**Published:** 2024-06-17

**Authors:** Dawei Wang, Mengyuan Zhang, Meng Gong, Ying-Hei Chui

**Affiliations:** 1 Wood Science and Technology Centre, University of New Brunswick, Fredericton, NB E3C 2G6, Canada; dwang12@unb.ca (D.W.); mzhang16@unb.ca (M.Z.); 2 Department of Civil and Environmental Engineering, University of Alberta, Edmonton, AB T6G 1H9, Canada

**Keywords:** trembling aspen, lumber, specific gravity, modulus of elasticity, tensile strength, grade yield

## Abstract

Trembling aspen (*Populus tremuloides*) is one of the major species within *Populus*, a predominant genus of hardwoods in North America. However, its utilization has been limited to pulp and paper or wood-based composite boards. This study aimed at evaluating the major physical and mechanical properties of trembling aspen lumber, with an ultimate objective of using this species to produce engineered wood products (EWPs). The testing materials consisted of 2 × 4 (38 mm × 89 mm) trembling aspen lumber pieces in lengths of 8, 10, and 12 feet (2.44, 3.05, and 3.66 m) with two visual grades, select structural (SS) and No. 2. Machine Stress-Rated (MSR), and longitudinal stress wave (LSW), edgewise third-point bending (EWB), and axial tension tests were conducted on the lumber. It was found that, (1) by increasing the maximum knot size by a half-inch from one-quarter inch, the minimum modulus of elasticity (MOE) measured using the MSR, the mean, and the fifth-percentile ultimate tensile strength (UTS) decreased by about 8.8%, 20.1%, and 29.8%, respectively. (2) Approximately 44% of the trembling aspen lumber met the 1450f-1.3E grade for MSR lumber, and 62% qualified for the 1200f-1.2E grade. (3) There was a great potential for manufacturing cross-laminated timber (CLT) of grade E3, with a rejection rate of about 29%. (4) The mean UTS and MOE values of the SS-grade trembling aspen lumber were 22.88 MPa and 9519 MPa, respectively, being 25.5% and 11.3% lower than that of Spruce–Pine–Fir (S-P-F) lumber. The fifth-percentile UTS and MOE values were 11.57 MPa and 7404 MPa, respectively, marking a decrease of 13.3% and 1.5% compared to the S-P-F lumber. (5) The oven-dried specific gravity (SG) of the trembling aspen wood was 0.40, which was about 3.5% larger than the value provided in the Wood Handbook.

## 1. Introduction

The *Populus*, a predominant genus of hardwoods in North America, includes several major species, with trembling aspen (*Populus tremuloides*) being one of the most significant. Other notable species in this genus include Bigtooth Aspen (*P. grandidentata*), Balsam Poplar (*P. balsamifera*), Eastern Cottonwood (*P. deltoides*), and Black Cottonwood (*P. trichocarpa*) [1]. The *Populus* genus, widely found in the northern hemisphere and uniquely characterized by its rapid growth, easy asexual reproduction, and diverse species, offers unmatched utility among temperate trees [2]. In the past, it was often the main resource species for making oriented strand board (OSB) and pulp production [3,4,5]. Subsequently, extensive research has focused on studying the genetics of *Populus* to develop enhanced hybrids [6,7]. Investigations have also been conducted on the mechanical properties of specific hybrid *Populus* clones for structural lumber applications [8,9,10,11]. In the 1990s, due to the boom of wood-based composite boards, *Populus* was widely and specifically cultivated as a suitable material. Canada’s forests, which cover 347.7 million hectares, are mainly composed of spruce (53.2%), poplar (11.6%), and pine (9.3%) [12]. The sustainable supply volumes for softwoods in Canada were set at 157.8 million m^3^, while, for hardwoods (primarily *Populus*), they were set at 56.4 million m^3^ in 2020 [13]. However, the actual harvest volumes in that year were as low as 115.6 million m^3^ for softwoods and 25.5 million m^3^ for hardwoods [14], indicating a surplus of hardwood resources and the need for an increase in the utilization. The Canadian government has planned to adjust the sustainable supply volumes in the future to effectively manage forest resources and balance harvesting levels, potentially benefiting from increased market demand for hardwoods [14,15]. These moves pave a path for the Canadian wood industry to increase the use of hardwood species such as trembling aspen in the manufacturing of high-value engineered wood products (EWPs) like cross-laminated timber (CLT), glue-laminated timber (GLT/Glulam), and wood I-Joists.

Kretschmann et al. [16] evaluated the structural properties of hybrid poplar by testing 2 × 4 (38 mm × 89 mm) lumber and found that 65% of the samples met the “No. 2 and better” grades. As for the Machine Stress-Rated (MSR) lumber, it had a yield of about 60% for a mechanical stress grade of 1450f-1.3E, and a yield of slightly over 33% for a grade of 1800f-1.5E. Their results underscored the potential of hybrid poplar for manufacturing structural EWPs in the wood industry, especially when paired with optimized management techniques. The rising popularity of EWPs has led to an increasing consideration of hardwood species like trembling aspen for structural applications, marking a departure from their traditional usage. The new generation of EWPs was developed to reduce the fibre requirement, which allows for the utilization of lower-quality fibre, thereby providing access to a wider range of wood species such as trembling aspen [17]. Gong et al. [18] studied the planar shear properties of hybrid CLT with hardwood lumber as the cross-layer, including trembling aspen, black spruce (*Picea mariana*), white birch (*Betula papyrifera*), and yellow birch (*Betula alleghaniensis*). They found that trembling aspen and birches exhibited relatively high resistance to planar shear stresses compared to black spruce, with the average planar shear modulus and strength being approximately 180 MPa and 3.00 MPa, respectively. They indicated that incorporating hardwoods as cross-layers in CLT could significantly enhance its planar shear performance. Wang et al. [19] investigated the planar shear properties in relation to the macroscopic characteristics of poplar (*P. deltoides cv. I-69/55*) wood, such as the annual ring orientation and the presence of pith. The presence of pith significantly negatively impacted the planar shear properties due to its lower density, with these properties increasing as the distance from the pith increased. Their findings also suggested that the poplar wood exhibited promising potential for the effective utilization in the cross-layers of advanced hybrid CLT, with an average planar shear modulus and strength of about 177 MPa and 2.24 MPa, respectively. In addition to CLT, Han et al. [20] assessed the use of poplar wood (*P. tomentosa Carr.*) for producing Glulam beams and evaluated the bending properties of these beams, made from preservative-treated alkaline copper quaternary (ACQ) and phenol-formaldehyde (PF) resin-impregnated poplar, in different laminate configurations using four-point static bending tests. They found that the preservative-treated, resin-reinforced beams significantly surpassed the untreated ones, showing an increase of 58.7% and 65.6% in the modulus of elasticity (MOE) and modulus of rupture (MOR), respectively, suggesting that poplar wood could be effectively utilized in the manufacturing of EWPs.

The overall objective of this research program aims to explore the feasibility of utilizing trembling aspen for making EWPs, thereby increasing the yield of this material, and expanding the range of products in the supply chain. To meet this, the major physical and mechanical properties should be evaluated first, which is the goal of this study.

## 2. Materials and Methods

### 2.1. Materials

In this study, 2 × 4 trembling aspen lumber was used, which was supplied by a sawmill located in the Hines Creek, Alberta, Canada. A total of 368 pieces were sampled, which were divided into two bundles in terms of the visual grades, selected structural (SS) and No. 2, of three lengths (8, 10, and 12 feet), as shown in Table 1. These trembling aspen lumber pieces were sawn from the logs of an average diameter of 8.5 inches (215.9 mm) at the small end. All of the lumber was kiln-dried at a temperature of 95 °C for 96 h until reaching the target moisture content (MC) of about 16%. The lumber was graded in accordance with “Standard Grading Rules for Canadian Lumber” [21] by an inspector from the Alberta Forest Products Association. All of the lumber specimens were wrapped and shipped to the Wood Science and Technology Centre, the University of New Brunswick, Fredericton, Canada, for further testing and analysis of their properties. It should be pointed out that the SS-grade 8-foot-long lumber was planned for making finger-jointed lumber, so the ultimate tensile strength (UTS) was not tested, but the MOEs of the lumber were tested in this study.

### 2.2. Methods

#### 2.2.1. Modulus of Elasticity (MOE)

Three non-destructive evaluation (NDE) methods were employed to measure the MOE of each lumber piece with different purposes for this research program, including the MSR, longitudinal stress wave (LSW), and edgewise third-point bending (EWB) tests. The MSR testing was conducted under the “Cook-Bolinder” machine (model: Tecmach Limited Stress Grading Machine System SG-AF), a modified laboratory mode by using the flatwise centre-point bending method to obtain the MOE along the pieces with the mean, maximum, and minimum values [22]. The MSR results were used to analyze the grade yield in this study. A commercial handheld stiffness grading device (model: MTG-820), developed by Brookhuis (Enschede, The Netherlands) and TNO (Delft, The Netherlands), was used for testing the average MOE of each lumber piece. This device recorded the stress wave speed and attenuation in each piece to receive the signal in terms of the natural frequency of the lumber under longitudinal vibration [23,24,25], which can be used to calculate the MOE (Equation (1)), as follows:

(1) $$MOE=4\rho \cdot {\left(\frac{f_{n}l}{n}\right)}^{2}$$

where *ρ* = density (kg/m^3^), *n* = mode number, *l* = length (mm), and *f_n_* = *n*^th^ natural frequency under longitudinal vibration (Hz). This method was used for the on-site sorting of the lumber in this research program.

The EWB testing, with two load heads positioned at each of the one-third points along the span, was conducted on the lumber according to ASTM D198 [26] to verify the MOE obtained using the above two approaches. The testing span-to-depth ratio was set at 17, with a test span of 1513 mm. The loading speed was 3 mm/min. Each test was terminated when the load reached 2 kN. The mid-span deflection was measured by two 50 mm linear variable differential transformers (LVDTs) on each side. The Forintek In-Grade Lumber Testing Procedure [27], developed by “Forintek Canada Corp.”, was followed in this study. This procedure is designed to standardize the testing of in-grade lumber and to provide a more accurate representation of the mechanical properties of full-size lumber now widely used by Canadian industries. The centre of each lumber piece for the EWB test was randomly selected following the “Forintek Procedure”, which was achieved by generating a random number and a random ruler. The maximum strength-reducing defect (MSRD), a method for recording the location and size of the largest defect in each lumber sample, was recorded within the designated span of each lumber piece. The MC of each piece was measured and recorded using a moisture metre (model: Wagner Orion 950) prior to testing.

#### 2.2.2. Ultimate Tensile Strength (UTS)

The axial tension testing was conducted on the lumber in accordance with ASTM D198 [26] using the Metriguard Testing Machine (model: Metriguard 401). The testing span varied due to the different lengths of the specimens used, ranging from 6 to 8 feet. Each grip was fixed at a length of 2 feet. A loading rate of 10 kN/min was set, allowing for specimen failure at least in approximately 4 min. The visually captured MSRD was positioned within the span and as close to the centre as possible. The failure load and mode(s) were recorded immediately after each test, while the failure load and maximum strength-reduction characteristics (MSRCs) were measured following the “Forintek In-Grade Lumber Testing Procedure” [27]. The MSRC measurement aimed at documenting the failure location and mode(s), and to identify the correlations between the maximum defects based on the MSRDs and the strength.

#### 2.2.3. Moisture Content and Specific Gravity (SG)

After the axial tension testing, a defect-free 1-inch-thick wood block for each lumber piece was cut near the location of failure for the determination of the MC and the oven-dried SG, following ASTM D4442 [28].

## 3. Results and Discussion

### 3.1. Physical and Mechanical Properties

#### 3.1.1. Moisture Content and Specific Gravity

The statistics of the different grades, lengths, MCs, and SGs are summarized in Table 2. The average MC of the trembling aspen lumber at testing was about 7.0%, and the average SG across all groups was approximately 0.40. It can be noted that the average SG of the trembling aspen lumber in this study was about 3.5% larger than the value published in the Wood Handbook (the value from the Wood Handbook was adjusted from green to oven-dried for comparison) [29,30]. The MC of each lumber piece at testing was used to convert the MOE and UTS values to those at an MC of 15% following the ASTM D1990 procedure [31] for further data analysis.

#### 3.1.2. Modulus of Elasticity

**Figure 1:**
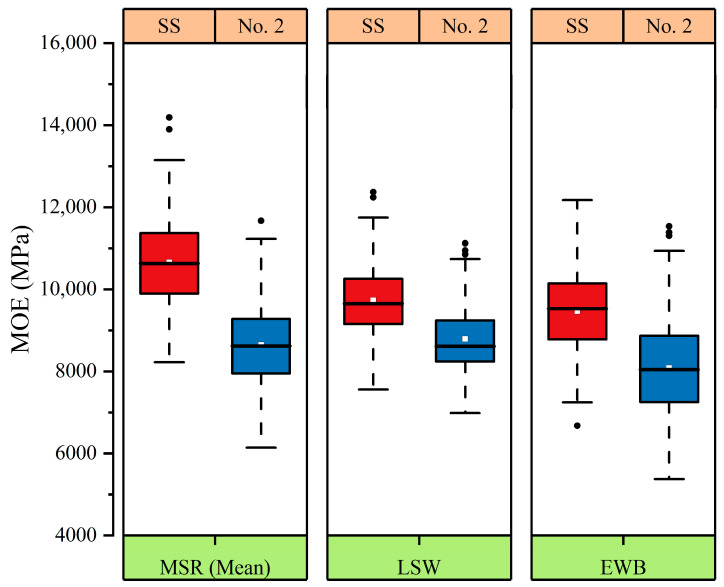
Mean MOE values between the two grades (SS-red, No. 2-blue) of three NDE methods used in this study.

The mean MOE values of the trembling aspen lumber measured by three methods are presented in Figure 1, ranging from 8032 MPa to 10,673 MPa. The values of the SS-grade lumber are all higher than those of the No. 2-grade lumber, with the MSR showing the biggest difference and the LSW having the least difference. The F-test at the 95% confidence level revealed a significant difference in the MOE results obtained by these three NDE methods, suggesting that the results of the MSR or LSW should be calibrated appropriately based on the outcomes from the EWB method. It should be pointed out that all of the MOE values measured in this study were in good agreement with those in other studies. Green and Evans [32] conducted the North American In-Grade Testing Program on 2 × 4 aspen–cottonwood lumber and found that the species had a mean MOE of 9860 MPa for the SS-grade lumber and 8818 MPa for that of the No.2-grade lumber at an MC of 15%. According to the American Forest & Paper Association (AF&PA) [33], the mean MOE of the aspen was measured at 7584 MPa for the SS-grade lumber and 6895 MPa for the No. 2-grade lumber at the MC of 12%. Kretschmann et al. [16] found that the 2 × 4 SS-grade and No. 2-grade hybrid poplar had a mean MOE of 9700 MPa and 8700 MPa, respectively, at the MC of 11%. The Wood Handbook [29] published a mean MOE value of 8100 MPa at the MC of 12% for trembling aspen.

**Figure 2:**
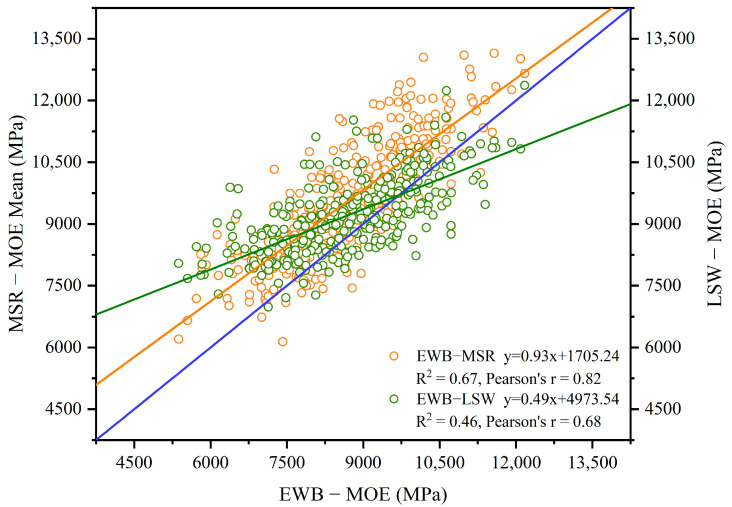
Relationships of the mean MOE values between the EWB and LSW (green)/MSR (orange) methods.

The statistical analysis revealed that the MOE measured by the MSR machine had a stronger linear correlation with those measured by the EWB method (r = 0.82) compared to the MOE measured by the LSW device (r = 0.68). This indicates that the MSR machine provided MOE measurements that were more closely aligned with the EWB method. The linear regression model was also conducted among the three methods. Figure 2 illustrates the relationship of the mean MOE values measured between the LSW/MSR and EWB methods. The blue line represents the 1:1 line. This analysis further demonstrated the comparative alignment of the MOE measurements obtained from the different testing methods. Although both R^2^ values were high, the MOE values tested by the MSR showed a better fit with the EWB method (R^2^ = 0.67) compared to the LSW (R^2^ = 0.46), indicating that the MSR provided a more accurate MOE model than the LSW. The results of the LSW/MSR tests were consistently higher than those obtained from the EWB. The purpose of this comparison was aimed at calibrating the MOE value measured via the LSW/MSR to the EWB method. The MSR technology is commonly used as a fast grading system on a production line producing EWPs such as wood I-Joists, and generated the mean, maximum, and minimum MOE values of the lumber. The information on the minimum MOE could be used as an indicator for the crosscutting of defects, most likely the MSRC, during the fabrication of finger-jointed lumber. The LSW technique was applied to lumber grading in this research program with the aim of pre-sorting the trembling aspen lumber in terms of the average MOE.

#### 3.1.3. Ultimate Tensile Strength and Failure Mode(s)

The tension testing results in Table 3 summarize that the SS-grade lumber has a mean UTS of 22.88 MPa, which is approximately twice as high as the mean UTS of the No. 2-grade lumber at 12.92 MPa, with a relatively low coefficient of variation (COV).

**Figure 3:**
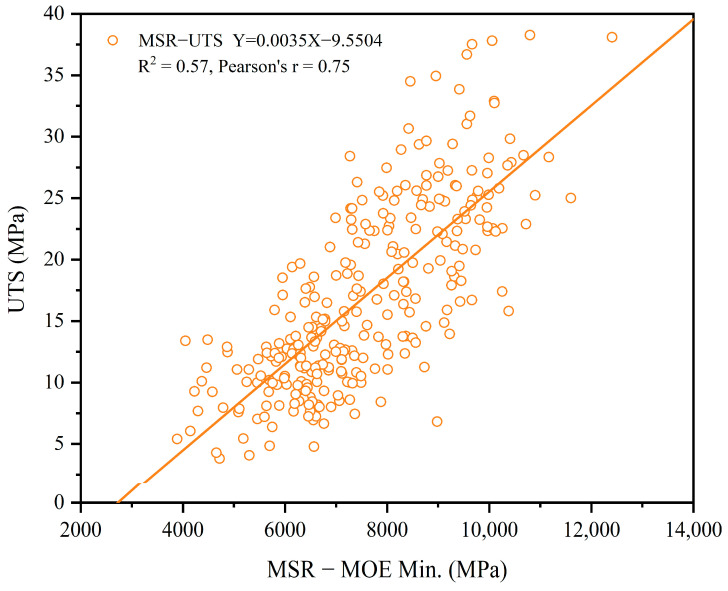
The relationship between UTS and MSR MOE_min_ (orange).

It should be pointed out that those pieces, which were broken within the machine grips, were culled from the data analysis, resulting in a rejection rate of about 12% for each group. Since the MSR MOE_min_ is closely related to a strength-reducing characteristic such as a knot, it shall have a close relationship with UTS as well, which is plotted in Figure 3. The high Pearson’s r-value of 0.75 further confirms this assumption. It also indicates the high R^2^ value in-between these two methods, which suggests that the MSR MOE_min_ is a reliable predictor for the UTS of trembling aspen lumber.

According to the MSRC, the same proportion of failure locations as predicted by the MSRD was 43.9% for the SS-grade lumber and 58.6% for the No. 2-grade lumber, respectively. The failure mode(s) of both the SS-grade and No. 2-grade trembling aspen lumber were mainly observed at the knot location during the axial tension testing, suggesting that strength of the trembling aspen lumber could be well predicted based on knot.

### 3.2. Effects of Lumber Length, Knot Size, and Species on Mechanical Properties

#### 3.2.1. Lumber Length

**Figure 4:**
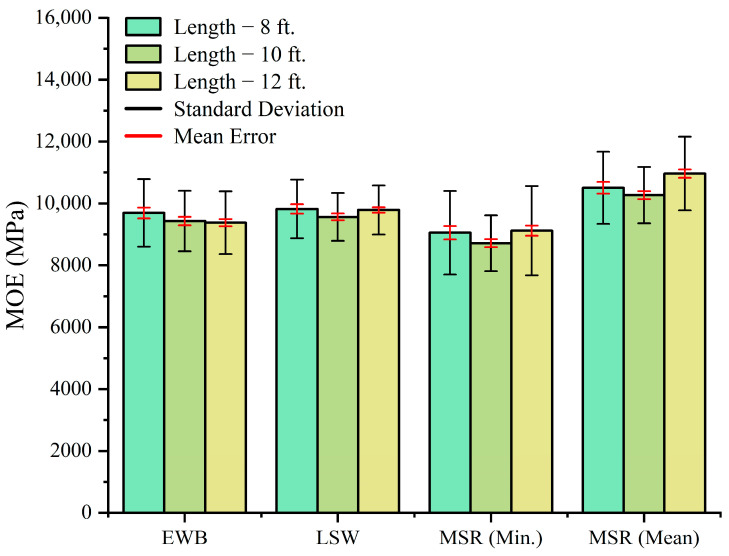
SS-grade trembling aspen mean MOE values measured by three NDE methods under different lengths.

Figure 4 presents the mean MOE values measured by the three NDE methods on the SS-grade trembling aspen lumber of three different lengths. The MOE values exhibited slight differences across the various length groups, under same testing method, ranging from 0.6% to 4.5%. Given the inherent variability due to the anisotropy of the wood material [34], the testing results in this study are considered reliable. It was concluded that, under the same testing method, the length effect does not significantly impact the outcomes.

#### 3.2.2. Knot Size

The strength is primarily determined by the size and location of the knots [35], making knots a crucial indicator for lumber grading systems [21]. Trembling aspen lumber was categorized as a “Northern Species” by the NLGA and was included in the softwoods category along with red cedar (*Thuja plicata*), red pine (*Pinus resinosa*), and Ponderosa pine (*Pinus ponderosa*) [21]. However, these rules may not be applicable to trembling aspen lumber due to its hardwood nature, showing unique growth characteristics associated with physical and mechanical properties. Compared to softwoods, dead knots and decay in trembling aspen are more easily identifiable [36,37], further affecting its properties. Therefore, a suitable grading rule for trembling aspen lumber should be developed by adapting the existing NLGA rules to accommodate its specific characteristics. To achieve this, re-classifying trembling aspen lumber only in terms of the maximum knot size (any-caused) were conducted in this study, based on the category as defined by the NLGA [21]. Four tiers were produced for the trembling aspen lumber, which are associated with the mechanical properties listed in Table 4. A clear relationship was found to exist between the maximum knot size and a given mechanical property, such as the MOE or UTS. As the maximum knot size increased by a half-inch from one-quarter inch, the MOE_min_ for the MSR, as well as the mean and fifth-percentile UTS, decreased by about 8.8%, 20.1%, and 29.8%, respectively.

**Figure 5:**
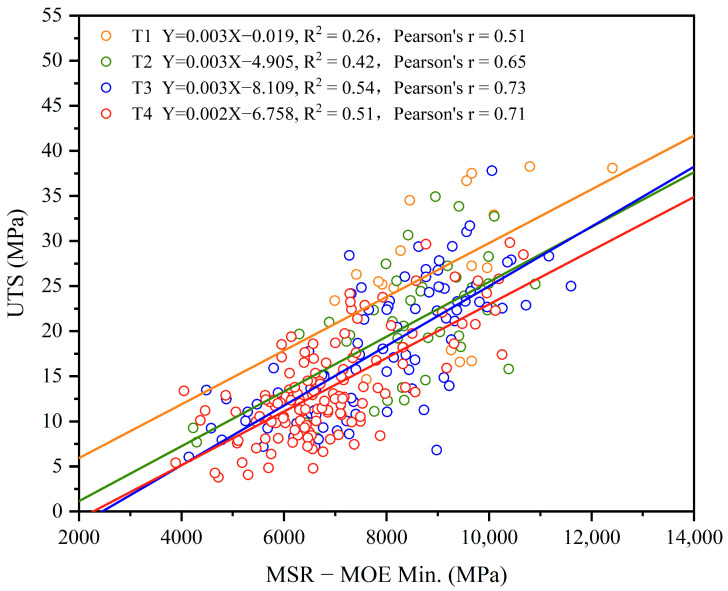
Four tiers (T1-orange, T2-green, T3-blue, T4-red) of relationship between the maximum knot size, MOE, and UTS.

The linear regression between the MSR (MOE_min_) and UTS was measured and is presented in Figure 5. The R^2^ values for T1 in Figure 5 are relatively low compared to the evident correlation observed for T2, T3, and T4, as might be expected due to the smaller sample sizes. The correlation between the maximum knot size and the four tiers, as shown in Figure 5, is evident, with R^2^ values ranging from 0.26 to 0.54, suggesting that the sub-grade approach is validated.

#### 3.2.3. Comparison of MOE and UTS with Spruce–Pine–Fir (S-P-F) Lumber

As a comparison, the UTS and MOE values of the 2 × 4 S-P-F [38] dimensional lumber are listed together with the trembling aspen lumber in Table 5. The MOE values for the SS-grade trembling aspen lumber, tested via the EWB method, were considered for a mixed combination of three different lengths. For the 8-foot-long lumber, the values of the MOE were considered, but not the UTS in this comparison, since the axial tension was not tested. The ratio was defined as the value of the trembling aspen lumber to that of the S-P-F lumber. It can be found that the MOE and UTS of the trembling aspen lumber are overall lower, regardless of the grade, than that of the S-P-F lumber, in particular for the UTS of the No. 2-grade lumber.

### 3.3. Grade-Yield Analysis

The MSR technology is widely used in sawmill production lines for quickly and accurately sorting the lumber grades for manufacturing EWPs, which requires appropriate machine settings. Smith and Chui [39] developed procedures for calculating the MSR machine settings and yield for each grade, represented by the percentage of lumber pieces falling into each grade. Despite NLGA SPS-2 [40] listing numerous MSR grades, only a few are commonly produced, namely, 1800f-1.6E, 1650f-1.5E, 1450f-1.3E, and 1350f-1.3E. Three combinations of MSR grades are considered in this analysis and are summarized in Table 6.

The grade yields for combinations 1 and 2 are moderate, while the grade yields for combination 3 are generally good. A total of 62% of all of the produced trembling aspen lumber qualified as grade 1200f-1.2E and above, with 44% reaching the grade of 1450f-1.3E. This confirmed the findings by Kretschmann et al. [16], who evaluated the “No. 2 and better” hybrid poplar at a grade of 1450f-1.3E with a yield of 60.5%.

In addition to the MSR lumber, the production of CLT using trembling aspen lumber was discussed as well with reference to PRG-320 [41], in which grade E1 was based on S-P-F lumber, while grade E3 included “Northern Species” such as trembling aspen lumber. Table 7 presents the results for the strength testing of the CLT panels. It can be noted that the yields for grade E1 of the trembling aspen lumber is 23%, existing only in the minor-strength direction. Grade E3 of the trembling aspen lumber constitutes 29% of the rejected material. It can be suggested that the trembling aspen lumber undergoes in-mill sorting in advance to reduce the rejection rate further and to allow for the sale of pieces that do not meet the CLT grade as visually graded lumber. The use of trembling aspen lumber for manufacturing CLT panels is uncommon, so it would be valuable to evaluate the yield of trembling aspen lumber after sorting it for use in CLT panels.

## 4. Conclusions

Based on the above data analysis and discussion, the following conclusions can be drawn:

1. It can be observed that, as the maximum knot size increased by a half-inch from one-quarter inch, the MOE_min_ measured using the MSR, as well as the mean and fifth-percentile UTS, decreased by about 8.8%, 20.1%, and 29.8%, respectively. It is recommended that the maximum knot size be incorporated as a primary factor in trembling aspen lumber sorting criteria, with the initial value of one-quarter inch being incremented by a half-inch.
2. Approximately 44% of the trembling aspen lumber met the 1450f-1.3E grade for MSR lumber, and 62% qualified for the 1200f-1.2E grade. Moreover, it exhibited significant potential for manufacturing E3-grade CLT, despite a rejection rate of about 29%.
3. The mean UTS and MOE values of the SS-grade trembling aspen lumber were 22.88 MPa and 9519 MPa, respectively, being 25.5% and 11.3% lower than those of the S-P-F lumber. The fifth-percentile UTS and MOE values were 11.57 MPa and 7404 MPa, respectively, marking a decrease of 13.3% and 1.5% compared to that of the S-P-F lumber.
4. The oven-dried SG of the trembling aspen wood was 0.40, about 3.5% larger than the value published in the Wood Handbook.
5. The length effect of the SS-grade trembling aspen lumber on the mean MOE values, with variations ranging from 0.6% to 4.5%, indicates no significant impact.

## Figures and Tables

**Table 1:** The dimension and quantity of the trembling aspen lumber sampled in Hines Creek, Alberta, Canada.

Dimension						Quantity (Pcs.)	
Length		Width		Thickness		SS Grade	No. 2 Grade
foot	mm	inch	mm	inch	mm		
8	2438	3.5	89	1.5	38	39	-
10	3048	3.5	89	1.5	38	49	-
12	3658	3.5	89	1.5	38	80	200

Note: “-” refers to no data.

**Table 2:** Property summary of the trembling aspen lumber tested.

Grade	Length (ft.)	Index	MC (%)	SG
SS	8	Count	39	39
		Mean	8.3	0.43
		COV (%)	13.5	7.4
	10	Count	49	49
		Mean	7.5	0.41
		COV (%)	8.5	8.5
	12	Count	80	80
		Mean	7.4	0.41
		COV (%)	4.4	6.9
No. 2	12	Count	200	200
		Mean	7.0	0.42
		COV (%)	7.3	8.9

Note: “COV” stands for coefficient of variation.

**Table 3:** UTS of trembling aspen lumber tested.

	SS Grade	No. 2 Grade
Count (Pcs.)	114	174
Failure at MSRD (Pcs.)	50	102
Reject (Pcs.)	15	26
Mean UTS (MPa)	22.88	12.92
COV (%)	29.1	38.9

Note: “MSRD” stands for maximum strength-reducing defect.

**Table 4:** Effect of the maximum knot size on the MSR MOE_min_ and UTS.

Index	Sub-Group			
	T1	T2	T3	T4
Max. Knot Size (any-caused) (inches)	<1/4 (6.35)	1/4–3/4 (19.05)	3/4–5/4 (31.75)	>5/4
MSR MOE_min_ (MPa)	9026	8286	7867	6842
Mean UTS (MPa)	26.87	20.27	17.94	13.60
Fifth-Percentile UTS (MPa)	14.16	9.12	6.74	4.87
Quantity (Pcs.)	19	45	89	135
Proportion (%)	6.6	15.6	30.9	46.9

Note: units in parentheses are in millimetres.

**Table 5:** MOE and UTS of trembling aspen and S-P-F lumbers.

Property	Grade	Sample Size (Pcs.)		Mean (MPa)			Fifth Percentile (MPa)		
		Aspen	S-P-F	Aspen	S-P-F	Ratio	Aspen	S-P-F	Ratio
MOE	SS	153	441	9519	10,730	0.89	7404	7520	0.98
	No. 2	174	440	8028	9490	0.85	5656	6090	0.93
UTS	SS	114	440	22.88	30.69	0.75	11.57	13.34	0.87
	No. 2	174	444	12.85	22.59	0.57	4.88	7.60	0.64

**Table 6:** MSR settings and grade yields based on MSR MOE_min_.

Combination	High-Grade			Low-Grade			Reject (%)
	Grade	Setting (MPa)	Yield (%)	Grade	Setting (MPa)	Yield (%)	
1	1650f-1.5E	9264	18	1450f-1.3E	7563	26	56
2	1650f-1.5E	9264	18	1350f-1.3E	7399	30	52
3	1450f-1.3E	7563	44	1200f-1.2E	6784	18	38

**Table 7:** Grade-yield analysis of trembling aspen lumber for manufacturing CLT based on PRG-320.

Grade	Major-Strength Direction			Minor-Strength Direction			Reject (%)
	Standard (MPa)	Setting (MPa)	Yield (%)	Standard (MPa)	Setting (MPa)	Yield (%)	
E1	11,700	-	-	9000	9001	23	77
E3	8300	8309	34	6500	6511	37	29

## Data Availability

The original contributions presented in the study are included in the article, further inquiries can be directed to the corresponding authors.
